# Widening inequalities in multimorbidity? Time trends among the working population between 2005 and 2015 based on German health insurance data

**DOI:** 10.1186/s12939-018-0815-z

**Published:** 2018-07-16

**Authors:** Juliane Tetzlaff, Jelena Epping, Stefanie Sperlich, Sveja Eberhard, Jona Theodor Stahmeyer, Siegfried Geyer

**Affiliations:** 1 Medical Sociology Unit, Hannover Medical School, Carl-Neuberg-Str.1, 30625 Hannover, Germany; 2 AOK Niedersachsen- Statutory Health Insurance of Lower Saxony, Hildesheimer Str. 273, 30519 Hannover, Germany

**Keywords:** Multimorbidity, Time trend, Social inequalities, Prevalence, Working population

## Abstract

**Background:**

Previous research has produced evidence for social inequalities in multimorbidity, but little is known on how these disparities change over time. Our study investigates the development of social inequalities in multimorbidity among the middle-aged and older working population. Special attention is paid to whether differing time trends between socio-economic status (SES) groups have taken place, increasing or decreasing inequalities in multimorbidity.

**Methods:**

The analyses are based on claims data of a German statutory health insurance company covering an observation period from 2005 to 2015. Multimorbidity prevalence risks are estimated using logistic generalized estimation equations (GEE) models. Predicted probabilities of multimorbidity prevalence are used to assess time trends in absolute social inequalities in terms of educational level, income, and occupational group.

**Results:**

The prevalence risks of multimorbidity rose among all SES groups and social gradients persist throughout the observation period, indicating significantly higher multimorbidity prevalence risks for individuals with lower SES. Widening absolute inequalities are found among men in terms of educational level and among women in terms of occupational groups.

**Conclusions:**

The increases in multimorbidity prevalence among the working population are accompanied by widening social inequalities, pointing towards a growing disadvantage for men and women in lower SES groups. The rising burden and the increasing inequalities among the working population stress the importance of multimorbidity as a major public health concern.

**Electronic supplementary material:**

The online version of this article (10.1186/s12939-018-0815-z) contains supplementary material, which is available to authorized users.

## Background

Within the past decade, multimorbidity has become a major public health concern challenging patients, health care providers, and health care systems. Multiple chronic conditions have a strong impact on the affected population, including poor health outcomes, lower quality of life, higher health care utilization and corresponding costs [1–3]. Research investigating trends in multimorbidity points towards increases in multimorbidity prevalence over time [4–6]. In the context of population ageing, these increases are expected to continue. However, studies analysing the development of multimorbidity over time are rare and little is known about time trends of social disparities in specific populations.

Previous research has produced evidence for social inequalities in multimorbidity, indicating a disadvantage in prevalence risks [2, 7] and earlier incidence during the life course for individuals with lower socio-economic status (SES). A Scottish study reported 10 to 15 years earlier onsets of multimorbidity in primary care patients living in the most deprived areas compared to those living in the most affluent [8]. The reasons for social inequalities in health are numerous and the underlying mechanisms are still not fully understood [9, 10]. Besides economic and occupation-related aspects, health behaviours and the level of health-related knowledge are widely accepted to foster health inequalities between SES groups [11, 12]. Research shows that this equally applies to multimorbidity as associations between risky health behaviours, such as physical inactivity, smoking, and high BMI, and multiple-chronic conditions are well documented [13, 14].

Over the last decades, growing income inequalities in Europe as well as in Germany are reported [15, 16]. These increasing social disparities raise the question whether all SES groups are affected by the prevalence increases similarly or whether time trends differ between groups, leading to growing health inequalities over time.

In order to keep the ratio of workers to non-workers in ageing populations constant, policies aim on growing labour force participation, especially among older and middle-aged adults [17]. As a result of the demographic change and an increased retirement age, the German working-age population ages rapidly [18]. Against this backdrop, it is important to examine health trends in middle-aged and older working population, taking into account that health might develop differently between SES groups. Multimorbidity represents a relevant health indicator as it is associated with declines in functional status, poor quality of life, and increased mortality risks [2, 3, 19–21]. Moreover, the presence of multiple conditions hampers the ability to meet job requirements and predicts lower labour force participation [22].

The present study aims to investigate the development of social inequalities in multimorbidity prevalence among the working population over time. Special attention is paid to the question whether differing time trends between SES groups have taken place, increasing or reducing health inequalities. The analyses are based on claims data of a large German health insurance provider which permit to include a broad range of chronic conditions. The data cover an observation period of 11 years. The study is guided by the following research questions:

1. Has the prevalence of multimorbidity increased among the working population over the time period from 2005 to 2015?
2. Are there social disparities in multimorbidity prevalence among the working population?
3. Are there differing time trends in multimorbidity prevalence between SES groups in the time period from 2005 to 2015?

## Methods

### Data

The analyses are based on claims data of the AOK Niedersachsen (AOKN), which is a large statutory health insurance provider in the federal state of Lower Saxony, Germany. Health insurance coverage is part of the welfare state-based health care system and is mandatory for all residents in Germany. Nearly 90% of the German inhabitants are insured by a statutory health insurance [23]. The insurance is based on premium payments and usually covers the full medical care at no or low additional costs.

The data were collected for accounting purpose and contain demographic and socio-economic information as well as in- and outpatient diagnoses, medications and all treatments covered by the insurance. With respect to sex and age, the AOKN population is comparable to the total population of Lower Saxony and Germany. However, the insurance population differs from the general population in terms of educational level and occupational positions as lower socio-economic groups are overrepresented [24].

### Socio-economic indicators

Employers in Germany are legally bound to report annually on salaries, qualification level, and occupational position to statutory health insurances. Thus, our data comprise information on annual gross income, educational level, and occupational group for employed insured individuals. In order to provide a deeper understanding of the underlying processes, each of these three SES indicators was analysed. Research indicates that each indicator is related to different causal processes influencing the health of individuals and that they are usually only moderately or weakly correlated to each other. This implies SES indicators cannot be used interchangeably [25, 26]. SES measures on income and occupational groups are based on previous studies using health insurance data of the AOKN [27].

*Educational level* was assessed using years of school education, indicating different degrees of school-leaving qualifications: 9 to 11 (low), and 12 to 13 years (high) of school education. *Income* is classified according to the average income in Germany in a given year reported by the German federal statistical office. Thus, income groups vary between years in absolute terms but are constant in relative terms, accounting for increasing income levels over time. Income was classified in three groups: < 40% (low), 40 to < 80% (middle), and 80% and above (high) of the annual average income from the German population. *Occupational groups* are based on an occupation classification system by Blossfeld [28]. The original system contains 12 groups. Occupations within the same group are comparable with regard to requirements on school-leaving qualification, vocational training, and professional activity. For the present study, these 12 groups were summarized into four: unskilled, skilled, specialists, and highly qualified. Specialists differ from skilled employees by higher qualification and higher level of decision latitude. Highly qualified employees usually have a university degree and demanding professional tasks. A detailed description on how the occupational groups were summarised and a listing of the original classification system by Blossfeld [28] can be found in Additional file 1.

As information on SES is only available for the working population, the following analyses are based on employed individuals of the years 2005 to 2015. The age range was restricted to individuals aged 40 to 65 years, because the prevalence of multimorbidity in our data is too low in younger age groups. As the dataset comprises the complete insurance population, most individuals are included over more than 1 year of observation (Table 1).

**Table 1:** Descriptive statistics of the number of insured individuals by gender (employed individuals)

	Men		Women	
	*N*	*%*	*N*	*%*
Year				
2005	239,079	7.7	165,299	7.6
2006	242,482	7.8	166,758	7.6
2007	249,280	8.0	171,261	7.8
2008	251,817	8.1	173,394	7.9
2009	253,742	8.1	177,021	8.1
2010	299,847	9.6	205,467	9.4
2011	309,946	9.9	214,543	9.8
2012	315,238	10.1	220,668	10.1
2013	316,387	10.2	223,123	10.2
2014	318,707	10.2	228,181	10.5
2015	322,003	10.3	238,129	10.9
Age group				
40–44	754,613	24.2	500,417	22.9
45–49	800,681	25.7	571,594	26.2
50–54	696,113	22.3	516,881	23.7
55–59	536,537	17.2	392,695	18.0
60–65	330,584	10.6	202,257	9.3
Educational level				
low	2,178,916	69.9	1,447,806	66.3
high	158,979	5.1	170,262	7.8
missing	780,633	25.0	565,776	25.9
Income				
low	104,879	3.4	428,606	19.6
middle	536,416	17.2	840,409	38.5
high	1,761,154	56.5	472,309	21.6
missing	716,079	23.0	442,520	20.3
Occupational group				
unskilled	960,740	30.8	898,156	41.1
skilled	1,464,937	47.0	502,155	23.0
specialists	285,572	9.2	599,088	27.4
highly qualified	109,784	3.5	85,249	3.9
missing	297,495	9.5	99,196	4.5
Multimorbidity	81,489	2.6	67,364	3.1
total number of observations	3,118,528	58.8	2,183,844	41.2
at least 2 years of observation	2,835,451	90.9	1,973,699	90.4

### Definition of multimorbidity

Previous studies indicate that a simple count of diagnosis codes is not sufficient if time trends in multimorbidity based on health insurance claims data are studied [29]. Changed diagnostic practices and increased sensibility of patients and physicians may foster earlier detection of diseases and increase the completeness of coded diagnoses over time [29]. Therefore polypharmacy was chosen as an additional criterion to diagnosis codes. Including polypharmacy shifted the focus to individuals requiring constant medical supervision and a higher degree of medical care and reduced the impact of changing coding practice that might have taken place over time.

The selection of diagnosis codes is based on 46 disease groups and risk factors included in the MultiCare Study [30], which covers a broad range of chronic conditions. Our data contain diagnosis codes according to ICD-10-GM. Haemorrhoids were excluded because the ICD-10 code changed during the study period leading to an implausible reduction in the number of coded diagnoses. A complete list of the ICD-10 codes used in this study is included in the supplementary material (Additional file 2). Data on all drugs covered by the statutory health insurance were used for defining polypharmacy. Drugs are coded according to the Anatomic Therapeutic Chemical Classification System (ATC) [31]. The drugs were differentiated on fourth precision level, which permits changes in medication within chemical subgroups. By taking into account only diagnoses and drugs appearing in at least two quarters of a given year, short-term medication and non-chronic conditions were excluded. In accordance with previous studies based on these data, multimorbidity was defined as having six or more coded chronic conditions and five or more drugs prescribed. More detailed information on multimorbidity measurement issues can be found in prior publications [29, 32].

### Statistical analyses

The development of multimorbidity prevalence and the effect of socio-economic factors were analysed using logistic regression models accounting for repeated observation of the same subjects over time. For dichotomous outcome variables, logistic generalized estimation equations (GEE) models are appropriate. GEE models account for the dependency of observations within individuals in different points in time. Population averaged logistic GEE models estimate the averaged effect of the independent variables on the outcome variable [33]. Therefore, the interpretation of the results is comparable to those of a logistic regression models without this dependency. Within GEE regression models, the adjustment for intra-subject correlation is achieved by assuming a particular correlation structure for the repeated measurement of the dependent variable. For our analyses first-order autoregressive correlation structures were chosen, assuming a decreasing intra-subject correlation with increasing distance between years of observation. As logistic GEE models require panel data, the regression analyses were restricted to individuals having more than 1 year of observation (about 90%) (Table 1).

Analyses on health inequalities can be conducted using measures of relative or absolute disparities [34]. Logistic interaction models (year*SES) were applied to estimate the relative increase or decrease in health disparities over time. Predicted multimorbidity prevalence probabilities were used to illustrate health inequalities in absolute terms. In order to predict these probabilities over time, predictive margins based on the logistic GEE analyses were estimated using postestimation commands. These estimates include all significant interaction terms (year*SES) regarding differing time trends between SES groups. If no significant interactions were found, predictive margins estimates are based on logistic GEE regression models without interaction terms.

The present study focuses on health inequalities in absolute terms as this approach allows for a straightforward analysis of time trends in multimorbidity burden of the different SES groups. The major advantage of using predicted probabilities rather than simple prevalence proportions lies in the adjustment for changes in the structure of the study population over time (e.g. age and SES) that can be made if predicted probabilities are used.

It has to be kept in mind that the estimated interaction terms illustrate changes in health disparities relatively to the baseline level of multimorbidity risks in the first year of observation within a specific SES group. The same relative increase in multimorbidity prevalence risks might result in different levels of absolute inequalities due to such differences at the baseline level. Thus, health disparities in relative and absolute terms might develop differently, depending on the multimorbidity risk level of the respective SES group.

## Results

The characteristics of the study population are displayed in Table 1. The data comprise a total of 4,809,150 observations based on 700,844 individuals with at least 2 years of observation over time. Due to the restriction to employed individuals, the data contain a higher share of men than women. While the educational level was similar in both genders, income and occupational status tended to be higher in men. The total proportion of multimorbidity cases was about 3% (Table 1). The proportion of multimorbidity prevalent individuals increased among all SES groups over time. This holds for men as well as for women (Table 2).

**Table 2:** Mulitmorbidity prevalence proportion (%) by SES, calendar year, and gender (employed individuals) (AOK Niedersachsen, Lower Saxony, Germany, 2005–2015)

		Educational level			Income				Occupational group				
		low	high	missing	low	middle	high	missing	unskilled	skilled	specialists	highly qualified	missing
Men	2005	1.4	0.7	1.3	3.5	1.8	1.2	1.2	1.5	1.0	1.5	0.9	1.5
	2006	1.6	0.7	1.5	3.7	2.0	1.5	1.3	1.8	1.2	1.7	0.9	1.8
	2007	1.8	0.9	1.8	2.9	2.0	1.8	1.5	2.1	1.4	1.9	1.1	2.0
	2008	2.3	1.1	2.2	3.4	2.4	2.2	1.9	2.6	1.7	2.3	1.3	2.4
	2009	2.5	1.3	2.4	3.4	2.6	2.4	2.2	2.9	2.0	2.5	1.6	2.6
	2010	2.6	1.4	2.4	3.8	2.7	2.4	2.3	3.1	2.2	2.5	1.5	2.5
	2011	2.8	1.5	2.7	3.8	3.0	2.6	2.5	3.1	2.6	2.7	1.8	2.8
	2012	3.0	1.8	2.9	3.8	3.0	2.9	2.7	2.8	3.0	3.2	2.1	2.8
	2013	3.4	2.0	3.1	5.3	3.6	3.1	2.9	3.1	3.3	3.5	2.6	2.9
	2014	3.7	2.3	3.3	6.1	3.9	3.4	3.2	3.4	3.7	3.7	3.1	3.0
	2015	4.0	2.4	3.4	4.9	3.9	3.8	3.2	3.5	3.9	4.0	3.2	3.2
Women	2005	2.0	0.7	1.9	2.6	1.7	1.8	1.9	2.2	1.8	1.5	1.0	1.9
	2006	2.3	0.9	2.1	2.7	2.1	2.0	1.9	2.5	2.0	1.7	1.1	2.1
	2007	2.5	0.9	2.4	2.6	2.4	2.3	2.1	2.7	2.2	1.9	1.3	2.3
	2008	2.9	1.2	2.8	3.0	2.8	2.7	2.6	3.2	2.6	2.3	1.5	2.5
	2009	3.1	1.2	3.1	3.1	3.0	2.9	3.0	3.5	2.8	2.5	1.6	2.6
	2010	3.2	1.4	3.0	3.2	3.1	2.9	2.7	3.7	2.8	2.4	1.8	2.4
	2011	3.3	1.5	3.3	3.4	3.2	3.1	2.9	3.9	2.9	2.6	2.2	2.5
	2012	3.5	1.7	3.4	3.4	3.3	3.5	3.2	4.1	3.2	2.7	2.6	2.7
	2013	3.8	1.9	3.8	4.0	3.5	3.6	3.4	4.4	3.5	3.0	2.9	2.6
	2014	4.0	1.9	3.9	4.2	3.8	3.6	3.5	4.6	3.7	3.1	3.0	2.6
	2015	4.2	2.3	4.1	4.4	3.9	4.0	3.6	4.8	4.0	3.2	3.2	2.7

The GEE regression models indicate a distinct annual increase in multimorbidity risks over time. This increase was more pronounced in men (9% per year) than in women (6% per year) as displayed in Table 3. In both genders, a clear effect of educational level on multimorbidity risks could be observed. Having higher school education reduced multimorbidity risks by 32% in males and 38% in females. With regard to income, another gradient could be found. Belonging to the highest income group led to a decrease of multimorbidity risks by 28% in males. In females, income effects were much smaller. The reduction in multimorbidity risk amounted 9% in the highest income group of women. Comparing occupational groups, a gradient in women was found, as multimorbidity risks decreased with rising level of classification. In contrast to unskilled employees, highly qualified women had a 21% lower risk of suffering from multimorbidity. Among men, a similar pattern could be observed with the exception of specialists, who showed no significant difference compared to unskilled men (Table 3).

**Table 3:** Logistic GEE-regression on multimorbidity prevalence risks by SES, age, and year, stratified for gender

		Men			Women		
		OR	95%-CI	*p*	OR	95%-CI	*p*
Educational level	low	1			1		
	high	0.68	0.63–0.74	< 0.001	0.62	0.57–0.67	< 0.001
	missing	0.87	0.84–0.90	< 0.001	0.94	0.91–0.98	0.002
Income	low	1			1		
	middle	0.83	0.79–0.86	< 0.001	0.92	0.89–0.94	< 0.001
	high	0.72	0.69–0.75	< 0.001	0.91	0.88–0.95	0.001
	missing	0.83	0.80–0.87	< 0.001	1.05	1.02–1.09	< 0.001
Occupational group	unskilled	1			1		
	skilled	0.88	0.86–0.91	< 0.001	0.91	0.88–0.94	< 0.001
	specialists	1.04	0.99–1.08	0.116	0.81	0.78–0.83	< 0.001
	highly qualified	0.83	0.77–0.88	< 0.001	0.79	0.73–0.86	< 0.001
	missing	0.78	0.75–0.82	< 0.001	0.63	0.58–0.68	< 0.001
Year		1.09	1.09–1.10	< 0.001	1.06	1.06–1.06	< 0.001
Age		1.15	1.14–1.15	< 0.001	1.13	1.12–1.13	< 0.001
Number of subjects		407,274			293,570		
Number of observations		2,835,451			1,973,699		
Wald Chi^2^ (*p*)		29,912.71 (df = 11) (< 0.001)			18,181.09 (df = 11)(< 0.001)		

OR odds ratio,95%-CI 95%-confidence interval, *p p*-value, df degrees of freedom

The interactions year*SES show the development of multimorbidity prevalence risks in relative terms within each SES group over time (Additional file 3). Significant interactions (*p* < 0.05) of year and SES were found for income groups (men and women), educational level (only women), and occupational groups (only men). All significant interactions point towards stronger increases of multimorbidity risks in higher than in lower SES groups. However, it has to be noted that the interaction terms are quite small compared to the general time trend and indicate only slight differences in time trends between SES groups (Additional file 3).

**Fig. 1:**
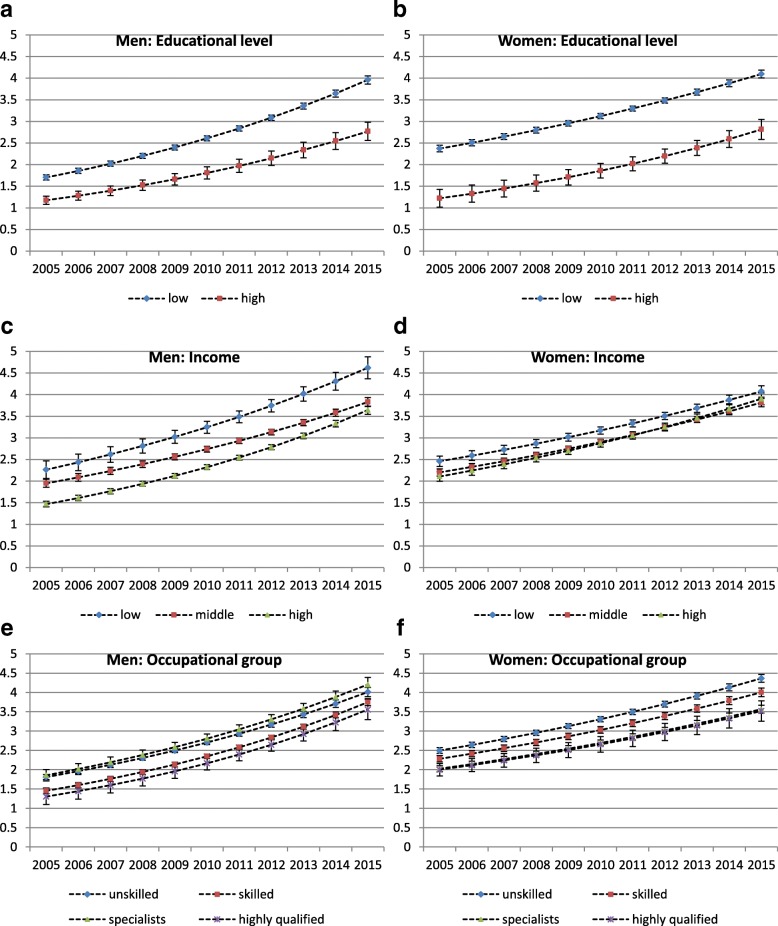
Predicted probabilities of multimorbidity prevalence (%) and 95%-confidence intervals by year and SES, stratified for gender: (**a**) Men: Educational level (**b**) Women: Educational level (**c**) Men: Income (**d**) Women: Income (**e**) Men: Occupational group (**f**) Women: Occupational group. Predicted probabilities are derived from logistic GEE-models including SES indicators educational level, income, and occupational group simultaneously. All analyses are controlled for age

The development of multimorbidity prevalence probabilities over time in absolute terms based on predictive margins stratified by gender and SES indicators is illustrated in Fig. 1. Between 2005 and 2015, probabilities between educational groups in males showed a rising (absolute, in terms of percentage points) difference over time (Fig. 1a), while absolute differences in females remained quite stable (Fig. 1b). Despite narrowing multimorbidity probabilities in middle and high income groups, the differences between the highest and the lowest income group among men remained constant (Fig. 1c). Among women, the differences between income groups were much smaller and no clear change in health inequality could be observed (Fig. 1d). In men, multimorbidity probabilities by occupational groups increased nearly parallel over time. As already indicated by the regression analyses, specialists had higher multimorbidity probabilities than skilled and highly qualified employees (Fig. 1e). Among women, absolute differences in multimorbidity probabilities between the highest and the lowest occupational group increased slightly over time, while probabilities of specialists and highly qualified women were nearly identical (Fig. 1f).

## Discussion

Our study shows that not all SES groups are equally affected by the observed increase in multimorbidity prevalence that has taken place over time. While multimorbidity prevalence rose among all SES groups, we found widening absolute inequalities in multimorbidity among the middle-aged and older working population. However, the evidence for growing inequalities is limited as increases in absolute difference in multimorbidity prevalence probabilities between groups are restricted to educational level among men and occupational group among women.

Our findings are in line with previous studies that reported increases in multimorbidity prevalence over time [4–6]. While the prevalence proportions among older individuals insured by the AOKN tend to be higher in women [29, 32], the general level of the predicted probability of multimorbidity prevalence of men and women in the working population aged 40 to 65 years is pretty similar.

Previous research has produced evidence for social inequalities in multimorbidity. The present study supports these findings as social gradients in multimorbidity had been found, indicating a disadvantage for lower SES groups. These inequalities were found for both genders and persisted throughout the observation period. With regard to educational level and occupational groups, no distinct gender differences in the strength of these gradients could be found. However, a much smaller income gradient in women than in men was observed which can most likely be explained by the generally lower income level of women. Against the backdrop of stronger increases in multimorbidity prevalence risks over time in men, our analyses show a rather strong increase in absolute health disparities in terms of educational level. In women, the growth of inequalities in multimorbidity was less pronounced and appeared only in differences between occupational groups. These findings indicate a slower progress of social disparities in multimorbidity in women than in men.

The estimated interaction terms indicate a tendency towards narrowing relative inequalities. However, this is not contradicting the widening absolute inequalities reported in this study. The interaction terms represent changes in health disparities within a specific SES group relatively to the level of multimorbidity risks in the first year of observation. Due to such differences at the baseline level, the same relative change in multimorbidity prevalence risks results in different amounts of absolute change. However, decreases in relative inequalities would have led to narrowing absolute inequalities if they would have been much stronger or would have been constant over a much longer time period than the observation period covered by the data.

### Strengths and limitations

Up to now, little is known about the development of health inequalities in multiple chronic conditions over time. Our study attempts to step into this gap by comparing time trends in multimorbidity between SES groups among the working population using population-based health insurance claims data. The data contain large case numbers and provide detailed information on diagnoses and medications that permit to include a wide range of chronic conditions. Furthermore, our data represent a complete insurance population and are therefore unaffected by health-related nonresponse, which could occur if ill health leads to reduced study participation in surveys [35]. With respect to age and sex, the insurance population is comparable to that of Germany but differs from the general population in terms of occupational groups and educational levels [24] which could lead to an overestimation of morbidity if crude measures are employed. This overrepresentation of individuals holding lower socio-economic positions is controlled for as all analyses performed are adjusted for SES.

As the analyses are restricted to the working population, early retirement and unemployment due to poor health may lead to an underestimation of multimorbidity prevalence if the health of working-age populations should be investigated. However, following the assumption that the impact of poor health on early retirement and unemployment is constant over time, the reported time trends in inequalities in multimorbidity remain unaffected.

The chosen multimorbidity measure is based on a definition that classifies individuals as suffering from multimorbidity, who have six or more chronic conditions and are treated with five or more drugs. This strict definition leads to rather low prevalence proportions among the middle-aged and older working population but is considered to be robust against changing coding practice that might have taken place over time as it shifts the focus to individuals requiring a higher degree of medical care [29, 32]. The application of a less restrictive definition would have led to higher prevalence proportions because less severe degrees of multimorbidity are expected to develop earlier in life but would have reduced the reliability of the results due to measurement issues.

The dataset used contains information on education, income, as well as on occupational position. As it is known, that these indicators measure different dimensions of determinants influencing health they cannot be used interchangeably [25, 26]. The socio-economic information included in our dataset allows to analyse health inequalities in terms of each of these three SES indicators simultaneously, which can provide a deeper understanding of the underlying processes.

## Conclusions

Using health insurance claims data, we found that time trends in multimorbidity among the working population differ between SES groups. This led to a growing disadvantage in men with lower educational levels and, to a lesser extent, women holding lower occupational positions. Thus, absolute social inequalities in multimorbidity are not only persisting but increasing over time. Keeping in mind that the presence of multiple chronic conditions hampers the ability to meet job requirements, growing inequalities in multimorbidity can be expected to foster both, the economic and the health disadvantage of individuals in lower SES groups. As policies aim on prolonging working lives, public health efforts have to focus on reducing these inequalities and on maintaining health among working populations up into the higher ages. In order to gauge the limits for further public health improvements, additional research is needed on how changes in lifestyles, prevention strategies, and working conditions have influenced the observed trends.

## Additional files

Additional file 1: Classification of occupational groups used in the study, based on Blossfeld’s classification system (Blossfeld 1987). (PDF 353 kb)

Additional file 2: Chronic conditions and ICD-10-GM codes used in the study. (PDF 278 kb)

Additional file 3: Logistic GEE-regression on multimorbidity prevalence risks by SES indicators, age, and calendar year stratified for gender, including interactions year*SES indicator (AOK Niedersachsen, 2005–2015). (PDF 500 kb)

## Abbreviations

AOKN: AOK Niedersachsen, Statutory Local Health Insurance of Lower Saxony
ATC: Anatomic Therapeutic Chemical Classification System
Logistic GEE regression: Logistic generalized estimation equations regression
SES: Socio-economic status

## References

[1] Hopman P, Heins MJ, Korevaar JC, Rijken M, Schellevis FG (2016). Health care utilization of patients with multiple chronic diseases in the Netherlands: differences and underlying factors. Eur J Intern Med.

[2] Marengoni A, Angleman S, Melis R, Mangialasche F, Karp A, Garmen A (2011). Aging with multimorbidity: a systematic review of the literature. Ageing Res Rev.

[3] Salive ME (2013). Multimorbidity in older adults. Epidemiol Rev.

[4] Dhalwani NN, O'Donovan G, Zaccardi F, Hamer M, Yates T, Davies M (2016). Long terms trends of multimorbidity and association with physical activity in older English population. Int J Behav Nutr Phys Act.

[5] Uijen AA, van de Lisdonk EH (2008). Multimorbidity in primary care: prevalence and trend over the last 20 years. Eur J Gen Pract.

[6] van Oostrom SH, Gijsen R, Stirbu I, Korevaar JC, Schellevis FG, Picavet HS, et al. Time trends in prevalence of chronic diseases and multimorbidity not only due to aging: data from general practices and health surveys. PLoS One. 2016; 10.1371/journal.pone.0160264. 10.1371/journal.pone.0160264 PMC4970764 27482903

[7] Violan C, Foguet-Boreu Q, Flores-Mateo G, Salisbury C, Blom J, Freitag M, et al. Prevalence, determinants and patterns of multimorbidity in primary care: a systematic review of observational studies. PLoS One. 2014; 10.1371/journal.pone.0102149. 10.1371/journal.pone.0102149 PMC4105594 25048354

[8] Barnett K, Mercer SW, Norbury M, Watt G, Wyke S, Guthrie B (2012). Epidemiology of multimorbidity and implications for health care, research, and medical education: a cross-sectional study. Lancet.

[9] Nutbeam D (2000). Health literacy as a public health goal: a challenge for contemporary health education and communication strategies into the 21st century. Health Promot Int.

[10] Pampel FC, Krueger PM, Denney JT (2010). Socioeconomic disparities in health behaviors. Annu Rev Sociol.

[11] Braveman P, Egerter S, Williams DR (2011). The social determinants of health: coming of age. Annu Rev Public Health.

[12] Lindström M, Kawachi I, Subramanian SV, Kim D (2008). Social Capital and Health-related behaviors. Social capital and health.

[13] Li J, Green M, Kearns B, Holding E, Smith C, Haywood A, et al. Patterns of multimorbidity and their association with health outcomes within Yorkshire, England: baseline results from the Yorkshire health study. BMC Public Health. 2016; 10.1186/s12889-016-3335-z. 10.1186/s12889-016-3335-z PMC4964308 27464646

[14] Wikstrom K, Lindstrom J, Harald K, Peltonen M, Laatikainen T (2015). Clinical and lifestyle-related risk factors for incident multimorbidity: 10-year follow-up of Finnish population-based cohorts 1982-2012. Eur J Intern Med.

[15] Piketty T, Saez E (2014). Inequality in the long run. Science.

[16] Statistisches Bundesamt, WZB. Datenreport 2016: Ein Sozialbericht für die Bundesrepublik Deutschland. Bonn: 2016. https://www.destatis.de/DE/Publikationen/Datenreport/Downloads/Datenreport2016.pdf?__blob=publicationFile. Accessed 23 Jan 2018.

[17] Vaupel JW, Loichinger E (2006). Redistributing work in aging Europe. Science.

[18] Statistische Ämter des Bundes und der Länder (2009). Demografischer Wandel in Deutschland.

[19] Fortin M, Lapointe L, Hudon C, Vanasse A, Ntetu AL, Maltais D (2004). Multimorbidity and quality of life in primary care: a systematic review. Health Qual Life Outcomes.

[20] Hsu HC (2015). Trajectories of multimorbidity and impacts on successful aging. Exp Gerontol.

[21] Nunes BP, Flores TR, Mielke GI, Thume E, Facchini LA (2016). Multimorbidity and mortality in older adults: a systematic review and meta-analysis. Arch Gerontol Geriatr.

[22] Smith P, Chen C, Mustard C, Bielecky A, Beaton D, Ibrahim S (2014). Examining the relationship between chronic conditions, multi-morbidity and labour market participation in Canada: 2000–2005. Ageing Soc.

[23] Statistisches Bundesamt. Sozialleistungen. Angaben zur Krankenversicherung (Ergebnisse des Mikrozensus). Wiesbaden: 2016. https://www.destatis.de/DE/Publikationen/Thematisch/Bevoelkerung/HaushalteMikrozensus/KrankenversicherungMikrozensus2130110159004.pdf;jsessionid=B212C632E42E8B604A2D004CA503FA7F.InternetLive1?__blob=publicationFile. Accessed 23 Jan 2018.

[24] Jaunzeme J, Eberhard S, Geyer S (2013). How "representative" are SHI (statutory health insurance) data? Demographic and social differences and similarities between an SHI-insured population, the population of lower Saxony, and that of the Federal Republic of Germany using the example of the AOK in lower Saxony. Bundesgesundheitsbl Gesundheitsforsch Gesundheitsschutz.

[25] Braveman PA, Cubbin C, Egerter S, Chideya S, Marchi KS, Metzler M (2005). Socioeconomic status in health research: one size does not fit all. JAMA.

[26] Geyer S, Hemstrom O, Peter R, Vagero D (2006). Education, income, and occupational class cannot be used interchangeably in social epidemiology. Empirical evidence against a common practice. J Epidemiol Community Health.

[27] Epping J, Muschik D, Geyer S (2017). Social inequalities in the utilization of outpatient psychotherapy: analyses of registry data from German statutory health insurance. Int J Equity Health.

[28] Blossfeld H-P (1987). Labor-market entry and the sexual segregation of careers in the Federal Republic of Germany. Am J Sociol.

[29] Tetzlaff J, Junius-Walker U, Muschik D, Epping J, Eberhard S, Geyer S (2017). Identifying time trends in multimorbidity—defining multimorbidity in times of changing diagnostic practices. J Public Health.

[30] Van den Bussche H, Scherer M (2011). Das Verbundvorhaben “Komorbidität und Multimorbidität in der hausärztlichen Versorgung” (MultiCare). Z Gerontol Geriatr.

[31] WHO Collaborating Centre for Drug Statistics Methodology (2015). Guidelines for ATC classification and DDD assignment. Oslo.

[32] Tetzlaff J, Muschik D, Epping J, Eberhard S, Geyer S (2017). Expansion or compression of multimorbidity? 10-year development of life years spent in multimorbidity based on health insurance claims data of lower Saxony, Germany. Int J Public Health.

[33] Twisk JW (2013). Applied longitudinal data analysis for epidemiology: a practical guide.

[34] Mackenbach JP, Kunst AE (1997). Measuring the magnitude of socio-economic inequalities in health: an overview of available measures illustrated with two examples from Europe. Soc Sci Med.

[35] Geyer S, Jaunzeme J, Swart E, Ihle P, Gothe H, Matusiewicz D (2014). Möglichkeiten und Grenzen von Befragungsdaten und Daten gesetzlicher Krankenversicherungen. Routinedaten im Gesundheitswesen: Handbuch Sekundärdatenanalyse: Grundlagen, Methoden und Perspektiven.

