# Supplementary material for: Widening inequalities in multimorbidity? Time trends among the working population between 2005 and 2015 based on German health insurance data
Source: Int J Equity Health. 2018 Jul 16;17:103. doi: 10.1186/s12939-018-0815-z (PMC6048702; doi:10.1186/s12939-018-0815-z)
Supplement: Supplementary file 1 — Classification of occupational groups used in the study, based on Blossfeld’s classification system (Blossfeld 1987). (PDF 353 kb) [file 12939_2018_815_MOESM1_ESM.pdf]

<u>Additional file 1</u>

Widening inequalities in multimorbidity? Time trends among the working population between 2005 and 2015 based on German health insurance data

Juliane Tetzlaff[1], Jelena Epping[1], Stefanie Sperlich[1], Sveja Eberhard[2], Jona Theodor Stahmeyer[2], Siegfried Geyer[1]

[1] Medical Sociology Unit, Hannover Medical School, Hannover, Germany

[2] AOK Niedersachsen- Statutory Health Insurance of Lower Saxony, Hannover, Germany

Additional file 1. Classification of occupational groups used in the study, based on Blossfeld's classification system (Blossfeld 1987)

Name of Occupational Group	Description of the Occupational Group	Examples	Summarised Classification used in the Study
Production			
Agricultural occupations (AGR)	Occupations with a dominant agricultural orientation	Farmers, agricultural workers, gardeners, workers in the forest economy, fishermen	*)
Unskilled manual occupations (EMB)	All manual occupations that showed at least 60 percent unskilled workers in 1970	Miners, rock breakers, paper makers, wood industry occupations, printing industry occupations, welders, unskilled workers, road and railroad construction workers	Unskilled
Skilled manual occupations (QMB)	All manual occupations that showed at most 40 percent unskilled workers in 1970	Glassblowers, bookbinders, typesetters, locksmiths, precision instrument makers, electrical mechanics, coopers, brewers	Skilled
Technicians (TEC)	All technically trained specialists	Machinery technicians, electrical technicians, construction technicians, mining technicians	Specialists
Engineers (ING)	Highly trained specialists who solve technical natural science problems	Construction engineering, electrical and engineers, production designers, chemical engineers, physicists, mathematicians	Highly qualified
Service			
Unskilled services (EDB)	All unskilled personal services	Cleaners, waiters, servers	Unskilled
Skilled Services (QDB)	Essentially order and security occupations as well as skilled service occupations	Policemen, firemen, locomotive engineers, photographers, hairservice dressers	Skilled
Semiprofessions (SEMI)	Service positions which are characterized by professional specialization	Nurses, educators, elementary school teachers, Kindergarten teachers	Specialists
Professions (PROF)	All liberal professions and service positions which require a university degree	Dentists, doctors, pharmacists, judges, secondary education teachers, university professors	Highly qualified
Administration			
Unskilled commercial and administrational occupations (EVB)	Relatively unskilled office and commerce occupations	Postal occupations, shop assistants, typists	Unskilled
Skilled commercial and administrational occupations (QVB)	Occupations with medium and higher administrative and distributive functions	Credit and financial assistants, foreign trade assistants, data processing operators, bookkeepers, goods traffic assistants	Specialists
Managers (MAN)	Occupations which control factors of production as well as functionaries of organizations	Managers, business administration, deputies, ministers, social organization leaders	Highly qualified

The original classification by Blossfeld (Source: Blossfeld H-P. Labor-Market Entry and the Sexual Segregation of Careers in the Federal Republic of Germany. American Journal of Sociology. 1987;93(1):89-118, Table 1: Classification of occupations) was summarised into for groups: unskilled, skilled, specialists, and highly qualified.

*)As Blossfeld's classification system does not distinguish between lower and higher qualified agricultural occupations, the occupations summarised into this original group were assigned to the four groups (unskilled, skilled, specialists, and highly qualified) according to the qualification level usually required for meeting the specific professional tasks.

The classification of occupational groups used in this study is based on the occupational classification system by Blossfeld 1987 (Blossfeld H-P. Labor-Market Entry and the Sexual Segregation of Careers in the Federal Republic of Germany. American Journal of Sociology. 1987;93(1):89-118). The original system contains 12 groups. The occupations within one group are comparable with respect to professional sectors, school or vocational training, and professional activities. Ignoring differences in the professional sector, these 12 groups were classified into four, summarising occupations with similar qualification levels and task complexity.

“Specialists” differ from “skilled” employees by higher qualification and higher decision-making latitude. “Highly qualified” individuals usually have a university degree and the occupation includes highly demanding tasks, e.g. management responsibilities.
