# Supplementary material for: Widening inequalities in multimorbidity? Time trends among the working population between 2005 and 2015 based on German health insurance data
Source: Int J Equity Health. 2018 Jul 16;17:103. doi: 10.1186/s12939-018-0815-z (PMC6048702; doi:10.1186/s12939-018-0815-z)
Supplement: Supplementary file 2 — Chronic conditions and ICD-10-GM codes used in the study. (PDF 278 kb) [file 12939_2018_815_MOESM2_ESM.pdf]

Additional file 2

Widening inequalities in multimorbidity? Time trends among the working population between 2005 and 2015 based on German health insurance data

Juliane Tetzlaff[1], Jelena Epping[1], Stefanie Sperlich[1], Sveja Eberhard[2], Jona Theodor Stahmeyer[2], Siegfried Geyer[1]

[1] Medical Sociology Unit, Hannover Medical School, Hannover, Germany

[2] AOK Niedersachsen- Statutory Health Insurance of Lower Saxony, Hannover, Germany

Additional file 2. Chronic conditions and ICD-10-GM codes used in the study

Chronic condition	ICD-10-GM Codes
Hypertension	I10-I15
Lipid metabolism disorders	E78
Chronic low back pain	M40-M45, M47, M48.0-M48.2, M48.5-M48.9, M50-M54
Severe vision reduction	H17-H18, H25-H28, H31, H33, H34.1-H34.2, H34.8-H34.9, H35-H36, H40, H43, H47, H54
Osteoarthrosis	M15-M19
Diabetes mellitus	E10-E14
Chronic ischemic heart disease	I20, I21, I25
Thyroid dysfunction	E01-E05, E06.1-E06.3, E06.5, E06.9, E07
Cardiac arrhythmias	I44-I45, I46.0, I46.9, I47-I48, I49.1-I49.9
Obesity	E66
Purine/pyrimidine metabolism disorders/Gout	E79, M10
Prostatic hyperplasia	N40
Lower limb varicosis	I83, I87.2
Liver disease	K70, K71.3-K71.5, K71.7, K72.1, K72.7, K72.9, K73-K74, K76
Depression	F32-F33
Asthma/COPD	J40-J45, J47
Noninflammatory gynecological problems	N81, N84-N90, N93, N95
Atherosclerosis/PAOD	I65-I66, I67.2, I70, I73.9
Osteoporosis	M80-M82
Renal insufficiency	N18-N19
Cerebral ischemia/Chronic stroke	I60-I64, I69, G45
Cardiac insufficiency	I50
Severe hearing loss	H90, H91.0, H91.1, H91.3, H91.8, H91.9
Chronic cholecystitis/Gallstones	K80, K81.1
Somatoform disorders	F45
Intestinal diverticulosis	K57
Rheumatoid arthritis/Chronic polyarthritis	M05-M06, M79.0
Cardiac valve disorders	I34-I37
Neuropathies	G50-G64
Dizziness	H81-H82, R42
Dementia	F00-F03, F05.1, G30, G31
Urinary incontinence	N39.3-N39.4, R32
Urinary tract calculi	N20
Anemia	D50-D53, D55-D58, D59.0-D59.2, D59.4-D59.9, D60.0, D60.8, D60.9, D61, D63-D64
Anxiety	F40-F41
Psoriasis	L40
Migraine/chronic headache	G43, G44
Parkinson's disease	G20-G22
Cancers	C00-C14, C15-C26, C30-C39, C40-C41, C43-C44, C45-C49, C50, C51-C58, C60-C63, C64-C68, C69-C72, C73-C75, C81-C96, C76-C80, C97, D00-D09, D37-D48
Allergies	H01.1, J30, L23, L27.2, L56.4, K52.2, K90.0, T78.1, T78.4, T88.7
Chronic gastritis/GERD	K21, K25.4-K25.9, K26.4-K26.9, K27.4-K27.9, K28.4-K28.9, K29.2-K29.9
Sexual dysfunction	F52, N48.4
Insomnia	G47, F51
Tobacco abuse	F17
Hypotension	I95

ICD-10-GM International Statistical Classification of Diseases and Related Health Problems, 10th Revision, German Modification. Selection of chronic conditions adapted from: van den Bussche H, Koller D, Kolonko T, Hansen H, Wegscheider K, Glaeske G, et al. Which chronic diseases and disease combinations are specific to multimorbidity in the elderly? Results of a claims data based cross-sectional study in Germany. BMC public

health. 2011;11:101. The original set of chronic conditions includes 46 groups. The group “haemorrhoids” was excluded due to changes of the ICD-10-GM code during the study period.
