# Supplementary material for: Widening inequalities in multimorbidity? Time trends among the working population between 2005 and 2015 based on German health insurance data
Source: Int J Equity Health. 2018 Jul 16;17:103. doi: 10.1186/s12939-018-0815-z (PMC6048702; doi:10.1186/s12939-018-0815-z)
Supplement: Supplementary file 3 — Logistic GEE-regression on multimorbidity prevalence risks by SES indicators, age, and calendar year stratified for gender, including interactions year*SES indicator (AOK Niedersachsen, 2005–2015). (PDF 500 kb) [file 12939_2018_815_MOESM3_ESM.pdf]

Additional file 3

Widening inequalities in multimorbidity? Time trends among the working population between 2005 and 2015 based on German health insurance data

Juliane Tetzlaff[1], Jelena Epping[1], Stefanie Sperlich[1], Sveja Eberhard[2], Jona Theodor Stahmeyer[2], Siegfried Geyer[1]

[1] Medical Sociology Unit, Hannover Medical School, Hannover, Germany

[2] AOK Niedersachsen- Statutory Health Insurance of Lower Saxony, Hannover, Germany

Additional file 3. Logistic GEE-regression on multimorbidity prevalence risks by SES indicators, age, and calendar year stratified for gender, including interactions year*SES indicator (AOK Niedersachsen, 2005-2015)

	Men			Women		
	OR	95%-CI	*p*	OR	95%-CI	*p*
Model 1: Interaction year*educational level						
**Year**	1.10	(1.09-1.10)	<0.001	1.06	(1.05-1.06)	<0.001
**Educational level**						
low	1			1		
high	0.62	(0.52-0.75)	<0.001	0.50	(0.42-0.60)	<0.001
missing	0.92	(0.87-0.98)	0.013	0.93	(0.87-0.99)	0.029
**Year*educational level**						
low	1			1		
high	1.01	(0.99-1.03)	0.225	1.03	(1.01-1.05)	0.005
missing	0.99	(0.98-1.00)	0.014	1.00	(0.99-1.01)	0.561
**Income**						
low	1			1		
middle	0.83	(0.79-0.86)	<0.001	0.92	(0.89-0.94)	<0.001
high	0.72	(0.69-0.75)	<0.001	0.91	(0.88-0.95)	<0.001
missing	0.83	(0.80-0.87)	<0.001	1.05	(1.02-1.09)	0.001
**Occupational group**						
unskilled	1			1		
skilled	0.88	(0.86-0.88)	<0.001	0.91	(0.88-0.94)	<0.001
specialists	1.03	(0.99-1.03)	0.125	0.81	(0.78-0.84)	<0.001
highly qualified	0.82	(0.77-0.88)	<0.001	0.79	(0.73-0.86)	<0.001
missing	0.78	(0.74-0.82)	<0.001	0.63	(0.58-0.68)	<0.001
**Age**	1.15	(1.14-1.15)	<0.001	1.13	(1.12-1.13)	<0.001
Number of subjects		407,274			293,570	
Number of observations		2,835,451			1,973,699	
Wald Chi^2 (*p*)		29904.02 (df=13) (<0.001)			18082.17 (df=13) (<0.001)	
Model 2: Interaction year*income						
**Year**	1.08	(1.07-1.09)	<0.001	1.05	(1.05-1.06)	<0.001
**Income**						
low	1			1		
middle	0.86	(0.77-0.95)	0.003	0.89	(0.84-0.95)	<0.001
high	0.64	(0.58-0.70)	<0.001	0.85	(0.79-0.92)	<0.001
missing	0.76	(0.69-0.84)	<0.001	1.00	(0.93-1.07)	0.935
**Year*income**						
low	1			1		
middle	1.00	(0.98-1.01)	0.501	1.00	(1.00-1.01)	0.270
high	1.02	(1.01-1.03)	0.005	1.01	(1.00-1.02)	0.019
missing	1.01	(1.00-1.03)	0.040	1.01	(1.00-1.02)	0.067
**Educational level**						
low	1			1		
high	0.68	(0.63-0.74)	<0.001	0.62	(0.57-0.67)	<0.001
missing	0.87	(0.84-0.90)	<0.001	0.94	(0.91-0.98)	0.002
**Occupational group**						
unskilled	1			1		
skilled	0.88	(0.86-0.91)	<0.001	0.91	(0.88-0.94)	<0.001
specialists	1.03	(0.99-1.08)	0.127	0.81	(0.78-0.83)	<0.001

highly qualified	0.82	(0.77-0.88)	<0.001	0.79	(0.73-0.86)	<0.001
missing	0.78	(0.74-0.82)	<0.001	0.63	(0.58-0.67)	<0.001
**Age**	1.15	(1.14-1.15)	<0.001	1.13	(1.12-1.13)	<0.001
Number of subjects		407,274			293,570	
Number of observations		2,835,451			1,973,699	
Wald Chi^2 (*p*)	29770.89 (df=14) (<0.001)			18193.65 (df=14) (<0.001)		
Model 3: Interaction year*occupational group						
**Year**	1.09	(1.08-1.09)	<0.001	1.06	(1.06-1.07)	<0.001
**Occupational group**						
unskilled	1			1		
skilled	0.80	(0.76-0.84)	<0.001	0.88	(0.82-0.94)	<0.001
specialists	1.03	(0.94-1.12)	0.555	0.85	(0.80-0.91)	<0.001
highly qualified	0.71	(0.60-0.84)	<0.001	0.68	(0.56-0.83)	<0.001
missing	0.89	(0.82-0.97)	<0.001	0.81	(0.71-0.91)	0.001
**Year*occupational group**						
unskilled	1			1		
skilled	1.01	(1.00-1.02)	0.091	1.01	(1.00-1.01)	0.205
specialists	1.00	(0.99-1.01)	0.676	1.00	(0.99-1.01)	0.682
highly qualified	1.02	(1.00-1.04)	0.044	1.02	(1.00-1.05)	0.067
missing	0.98	(0.97-0.99)	<0.001	0.96	(0.94-0.97)	<0.001
**Educational level**						
low	1			1		
high	0.68	(0.63-0.74)	<0.001	0.62	(0.57-0.67)	<0.001
missing	0.87	(0.84-0.90)	<0.001	0.94	(0.91-0.98)	0.002
**Income**						
low	1			1		
middle	0.83	(0.79-0.86)	<0.001	0.92	(0.89-0.94)	<0.001
high	0.72	(0.69-0.75)	<0.001	0.91	(0.88-0.95)	<0.001
missing	0.83	(0.80-0.87)	<0.001	1.05	(1.02-1.09)	0.001
**Age**	1.14	(1.14-1.15)	<0.001	1.13	(1.12-1.13)	<0.001
Number of subjects		407,274			293,570	
Number of observations		2,835,451			1,973,699	
Wald Chi^2 (*p*)	29951.4 (df=14) (<0.001)			18287.8 (df=14) (<0.001)		

OR odds ratio,95%-CI 95%-confidence interval, *p* p-value, df degrees of freedom
